# Growth performance and meat quality of meat-type guinea fowl fed different commercial diets

**DOI:** 10.5194/aab-64-325-2021

**Published:** 2021-07-28

**Authors:** Justyna Batkowska, Kamil Drabik, Małgorzata Karwowska, Umair Ahsan, Ifrah Raza, Agnieszka Adamczuk, Beata Horecka

**Affiliations:** 1 Institute of Biological Basis of Animal Production, University of Life Sciences in Lublin, 13 Akademicka St., 20-950 Lublin, Poland; 2 Department of Meat Technology and Food Quality, University of Life Sciences in Lublin, 8 Skromna St., 20-704 Lublin, Poland; 3 Department of Plant and Animal Production, School of Food, Agriculture and Livestock, Burdur Mehmet Akif Ersoy University, İstiklal Campus, Burdur 15030, Turkey; 4 Department of Animal Nutrition and Nutritional Diseases, Faculty of Veterinary Medicine, Aydın Adnan Menderes University, Işıklı, Aydın 09016, Turkey; 5 Institute of Agrophysics, Polish Academy of Sciences, 4 Doświadczalna St., 20-290 Lublin, Poland

## Abstract

The aim of study was to assess the growth performance, meat quality, and fatty acid composition of meat-type guinea fowl fed balanced commercial diets under two different feeding programs, similar to those for slaughter turkeys and broiler chickens, respectively. A total of 80 4-week-old meat-type guinea fowl divided into two groups (four replicates per group; 10 birds in each replicate) were raised for 14 weeks. One group received commercially available diets in a three-phased program (TM group), whereas the other group was fed commercial diets in a two-phased program (CM group). Growth-performance-related traits were recorded. At the end of rearing (14 weeks of age), eight birds from each group were slaughtered. Carcass yield and technological traits of meat (pH, color, water-holding capacity, natural and thermal loss, tenderness, fatty acid profile) were analyzed. Groups did not differ in terms of body weight as well as carcass yield and characteristics. There was no difference in meat quality and the fatty acid profile of breast and thigh meat of guinea fowl from TM and CM groups. The findings of this study suggest that both commercial diets (for broiler chickens and turkeys) can be used in meat-type guinea fowl rearing. Due to the lower price of diets fed to the CM group and the lack of significant variation in meat quality traits, its use seems to be more justified from an economic point of view.

## 1 Introduction

Guinea fowl is an indigenous species of African birds, although their adaptability has contributed to the spread of their breeding worldwide (Agwunobi and Ekpenyong, 1990). Variability of phenotypic features, availability of different color varieties (Agbolosu et al., 2014; Panyako et al., 2016), in addition to attractiveness of guinea fowl in terms of appearance, behavior, and obtained raw materials made them very popular among small-scale farmers (Moreki and Seabo, 2012). A recent study showed that guinea fowl is an important source of animal protein due to their high availability and relatively low market price (Obike et al., 2011).

In general, guinea fowl is used for laying purposes. During the season, the bird lays up to 190 eggs, relatively a higher production that may last for even 2 to 3 years (Gwaza and Elkanah, 2017). There is some diversification in laying characteristics between the bird's varieties (Onunkwo and Okor, 2015); however, guinea fowl eggs are noteworthy for their thick shell, high proportion of yolk (Alkan et al., 2013), high content of vitamins and trace elements (Bashir et al., 2015), and longer, in comparison to chicken eggs, shelf life (Ayorinde, 1991). Eggs are used for both direct consumption and processing (Agbolosu et al., 2014). Guinea fowl meat is described by a darker color and a unique flavor. It has a proper texture that is attributed to its white muscle fibers, which correspond in quantity to the muscles of chickens but in size to those of geese (Bernacki et al., 2012a). Guinea fowls are characterized by relatively higher slaughter performance (Ebegbulem and Asuquo, 2018) good ratios of valuable parts in the carcass as well as satisfactory sensory properties of meat (Kyere et al., 2020). Additionally, the nutritional value of guinea fowl meat distinguishes it from other poultry species due to higher protein and lower fat content (Ayorinde, 1991).

Several factors influence the quality and quantity of meat production of guinea fowl. Baeza et al. (2001) indicated the importance of genotype (variety) of guinea fowl and their sex, while Yamak et al. (2018) also mentioned age and husbandry conditions important as qualities determinant of meat. However, production results of these birds are constantly influenced by the quality and nutritional value of the feed. Under natural conditions, guinea fowl take up a variety of plant and animal feed, which indicates their adaptation to various sources of protein and energy. In countries where guinea fowl are a popular species of meat-type poultry, certain feeding norms are applied (Yildirim, 2012) that allow for the standardization of compound feed. However, in many countries, these birds are treated as an attraction and possibly used to obtain eggs mostly for self-supply of the farm despite being raised frequently on small-scale farms.

The supplying of guinea fowl carcasses is very limited, although consumers declare an interest in them. This may be attributed to no established nutrient requirements of guinea fowl in terms of protein, energy, amino acids, and other micro- and macro-nutrients, as well as there being no commercial balanced feed mixtures for these birds on the market. However, mixtures are widely available for the other meat-type birds, varying both in nutritive value and price. Therefore, the aim of this study was to assess the performance and meat quality of meat-type guinea fowls fed with commercial diets under the dedicated phases of feeding programs.

## 2 Material and methods

### 2.1 Bird rearing

According to Polish legislation the Directive 2010/63/EU implemented on the protection of animals used for scientific purposes (European Parliament and the Council of the European Union, 2010), research performed on animal raw material or as normal, production cycle are treated as non-experimental agricultural practices (Chapter I, article 1, point 5) and does not need ethical committee approval. At the same time, the birds were maintained in accordance with current legislation with regard to environmental conditions and stocking density.

A total of 80 4-week-old meat-type guinea fowl (French commercial set) were divided into two groups, each consisting of four replicates of 10 birds in each replicate. The birds were reared in a deep litter system. One group was fed commercial diets in a three-phased feeding program (TM group, starter 0–6 weeks, grower 7–12 weeks, finisher >13 weeks of age) similar to that of the slaughter turkeys' feeding program. The second group received commercial diets in a two-phased feeding program (CM group, starter 0–3 weeks, grower >4 weeks until slaughter) similarly to that of broiler chickens. The nutritional values of all diets are shown in Table 1.

**Table 1:** The composition of feed mixture dedicated for turkeys and chickens depending on the birds' age.

Item	Three-phased diets (TM group)			Two-phased diets (CM group)	
	Age of birds			Age of birds	
	0–6 weeks	7–12 weeks	>13 weeks	0–3 weeks	>4 weeks
Crude protein (%)	24.30	19.70	15.90	19.00	17.40
Crude fat (%)	4.10	4.00	3.80	3.80	5.10
Crude fiber (%)	3.20	3.50	3.60	3.70	4.10
Crude ash (%)	7.10	5.60	4.50	5.30	4.40
Lysine (%)	1.54	1.18	0.90	1.12	1.00
Methionine (%)	0.60	0.45	0.37	0.46	0.40
Calcium (%)	1.30	1.00	0.80	0.90	0.60
Phosphorus (%)	0.68	0.54	0.37	0.48	0.40
Sodium (%)	0.16	0.14	0.15	0.16	0.16
Selenium ($\mathrm{mg}\;{\mathrm{kg}}^{-1}$)	0.70	0.40	0.30	0.30	0.30
Iron ($\mathrm{mg}\;{\mathrm{kg}}^{-1}$)	88.00	48.00	40.00	40.00	34.00
Iodine ($\mathrm{mg}\;{\mathrm{kg}}^{-1}$)	2.00	1.00	1.00	1.00	1.00
Zinc ($\mathrm{mg}\;{\mathrm{kg}}^{-1}$)	166.00	90.00	75.00	75.00	63.00
Manganese ($\mathrm{mg}\;{\mathrm{kg}}^{-1}$)	133.00	72.00	60.00	60.00	51.00
Copper ($\mathrm{mg}\;{\mathrm{kg}}^{-1}$)	33.00	18.00	15.00	15.00	13.00
Vitamin A ($\mathrm{IU}\;{\mathrm{kg}}^{-1}$)	24 500.00	12 000.00	10 000.00	10 000.00	8500.00
Vitamin E ($\mathrm{IU}\;{\mathrm{kg}}^{-1}$)	105.00 $\mathrm{mg}\;{\mathrm{kg}}^{-1}$	30.00	25.00	25.0 0	21.00
Vitamin D${}_{3}$ ($\mathrm{IU}\;{\mathrm{kg}}^{-1}$)	6100.00 $\mathrm{IU}\;{\mathrm{kg}}^{-1}$	3100.00	3000.00	3000.00	2500.00
6-Phytase EC ($\mathrm{FTU}\;{\mathrm{kg}}^{-1}$)	1045.00	498.00	995.00	1005.00	503.00
Endo-1,4-beta xylanase ($U\;{\mathrm{kg}}^{-1}$)	1592.00	1592.00	1592.00	1608.00	2412.00
Monensin sodium/salinomycin ($\mathrm{mg}\;{\mathrm{kg}}^{-1}$)${}^{*}$	100.00	100.00	–	70.00	60.00

${}^{*}$ Coccidiostat withdrawn 14 d before slaughter.

### 2.2 Meat quality

All the birds were weighed individually on a weekly basis and feed intake was recorded. Based on this, the feed conversion ratio (FCR) was estimated. At the end of 14th week, eight birds were randomly selected and slaughtered from each group. Birds were subjected to feed restriction 8 h prior to slaughter. Birds were slaughtered (in a commercial poultry abattoir) by decapitation following electrical stunning using an electric current of 45 mA. The whipping procedure was in line with EU regulation no. 1099/2009 of 24 September 2009 (Council of the European Union, 2009) on the protection of animals at the time of slaughtering. After plucking and evisceration, the carcasses were chilled by air method (0 ${}^{\circ }C$, 4 h). Following this, parts of carcasses were extracted during the dissection (Hahn and Spindler, 2002): breast muscles, thighs, drumsticks, wings, and trunk, as well as the left breast and femoral muscles were taken for further analysis. Edible giblets (heart, liver, gizzard) and parts were weighed to assess the organs and part yields.

The evaluation of meat quality included pH measurement (15, 60 min, and 24 h after slaughter) with the use of pH meter CP-251, water-holding capacity according to Grau and Hamm (1953) method, natural drip loss (Lundstrom and Malmfors, 1985), and thermal (cooking) loss (Yang et al., 2006). Cuboid cores (1cm×1cm×2cm of edge length) were cut from the heat-treated muscles, parallel to the longitudinal orientation of the muscle fibers. Warner–Bratzler shear force was determined using a texture analyzer TA-XT plus (Stable Micro Systems Ltd., Surrey, UK) equipped with a V-shaped blade. Samples were shorn at a crosshead speed of 2 $\mathrm{mm}\;s^{-1}$.

Color parameters (Commision Internationale de l'Eclairage – CIE $L^{*}$, $a^{*}$, and $b^{*}$) were measured twice: on freshly cut muscle surfaces (after carcass cooling) and on heat-treated samples using an X-Rite Color®Premiere 8200 spectrophotometer (X-Rite Inc., Michigan, USA). The thickness of the samples was at least 10 mm. The instrumental conditions were a 25.4 mm diameter area aperture. The measurement was carried out in the range of 360–740 nm. The illuminant D65 and a 10${}^{\circ }$ standard colorimetric observer was used as a source of light. A white standard was used as a reference source with a specification of $L^{*}=95.87$, $a^{*}=-0.49$, $b^{*}=2.39$. The results were expressed in units of the CIELAB (Commision Internationale de l'Eclairage, 1978) system, for which the distinctions reflect the following, respectively:

- $L^{*}$ – color brightness, generally adopts positive values and can take values from 0 for an extremely black body and to 100 for a perfectly white body;
- $a^{*}$ – chromaticity in the red–green range; it shows red if it is positive, green if it is negative;
- $b^{*}$ – yellow–blue chromaticity; it shows yellow if it is positive, blue if it is negative.

The change of color parameters of samples (before and after heat treating) was calculated according to the equation and interpreted based on scale given below (Clydesdale, 1976):

1 $$\Delta E=\sqrt{{\left(\Delta L\right)}^{2}+{\left(\Delta a\right)}^{2}+{\left(\Delta b\right)}^{2}}.$$

The color change scale is as follows:

- 0<ΔE<1 – the observer does not see any difference,
- 1<ΔE<2 – only an experienced observer can see the difference,
- 2<ΔE<3.5 – the difference is also noted by the non-experienced observer,
- 3.5<ΔE<5 – the observer notes a clear color difference, and
- 5<ΔE – the observer gets the impression of two different colors.

### 2.3 Fatty acid profile

The fatty acid profile of breast and thigh meat was analyzed using the gas chromatography according to PN-EN ISO 5508: 1996 and PN-EN ISO 5509: 2001 using a Varian 450-GC gas chromatograph fitted with a flame ionization detector (FID). Injector and detector temperatures were 250 and 300 ${}^{\circ }C$, respectively. After injection, the column temperature was programmed to increase to 200 ${}^{\circ }C$ for 10 min, subsequently increased to 240 ${}^{\circ }C$ at the rate of 3 ${}^{\circ }C\;\min^{-1}$. Then, the column temperature was held at the final temperature for 4 min. Helium was used as a carrier gas (3 $\mathrm{mL}\;\min^{-1}$).

Based on the proportions of particular fatty acids and their groups, the following indexes were calculated: PI – peroxidation index (Arakawa and Sagai, 1986), AI – atherogenic index and TI – thrombogenic index (Ulbricht and Southgate, 1991), DFA – desirable fatty acid (Medeiros et al., 2014), HSFA – hypercholesterolaemic saturated fatty acid (Renna et al., 2012), and h / H – hypocholesterolemic / hypercholesterolemic ratio (Domaradzki et al., 2019). Additionally, the average carbon chain length (ACL) was estimated on the base of the number of carbon atoms in the determined fatty acids.

The obtained data were statistically analyzed using the SPSS 24.0 statistical package (IBM Corp., 2016) The groups were compared using a t test.

## 3 Results

### 3.1 Growth performance and carcass yield

Growth-performance-related traits of guinea fowl are presented in Table 2. The body weight of birds was similar during the whole rearing period regardless of the diets. Slightly better body weight gain was registered in the TM group than the CM group; however, these differences were not confirmed statistically. The feed conversion ratios were also similar in both groups except for period between eighth and ninth weeks, which was considerably greater in the TM group. However, it is impossible to explain this relation logically; it seems rather incidental. At the end of experiment, the TM group had slightly greater body weight. However, from an economic point of view, the difference was not statistically confirmed.

**Table 2:** Growth performance of guinea fowl in response to diets based on nutrient specifications for three- or two-phased diets (mean±SEM).

Age (weeks)	Body weight (g)			Age (weeks)	Feed conversion ratio (kg kg${}^{-1}$)		
	TM	CM	P value		TM	CM	P value
4	411±11.7	403±11.3	0.609	4–5	3.15±0.29	3.31±0.31	0.380
5	610±11.2	589±13.2	0.247	5–6	3.59±0.25	3.26±0.24	0.226
6	842±14.9	832±17.1	0.690	6–7	3.80±0.22	3.97±0.32	0.360
7	1109±14.3	1015±19.2	0.150	7–8	4.58±0.31	4.79±0.24	0.336
8	1296±18.8	1252±25.7	0.171	8–9	5.10±0.30	4.08±0.24	0.031
9	1536±45.9	1446±27.7	0.111	9–10	5.78±0.37	5.57±0.62	0.410
10	1641±29.6	1667±26.6	0.509	10–11	6.16±0.45	5.86±0.41	0.348
11	1768±31.6	1820±32.3	0.262	11–12	5.73±0.29	6.17±0.70	0.325
12	1968±29.7	1884±40.1	0.114	12–13	6.35±0.37	6.95±0.41	0.199
13	2125±44.9	2028±32.7	0.095	13–14	5.28±0.38	5.29±0.35	0.499
14	2291±46.9	2166±42.5	0.060	Total mean	4.79±0.03	5.11±0.03	0.000

TM – group fed with feed mixture for three phases, similar to slaughter turkeys, CM – group fed with feed mixture dedicated for two phases, similar broiler chickens.

There were no regular relations between groups in the case of feed conversion ratio. Mostly, the group fed the CM diet needed more feed per 1 kg of body weight gain that was reflected in overall growth period, whereas birds from the TM group utilized less feed per 1 kg of body weight gain. The difference amounted to 6.26 % and was statistically significant.

**Table 3:** Relative carcass, organs, and cut yields of guinea fowl in response to diets based on nutrient specifications for three- or two-phased diets (mean±SEM).

Item	Relative carcass and organ yields		
	(% proportion of body weight)		
	TM	CM	P value
Slaughter weight	2385±84.47	2302±82.68	0.581
Carcass yield	76.64±0.93	78.77±0.92	0.855
Liver	2.16±0.33	2.30±0.25	0.524
Heart	0.64±0.02	0.64±0.04	0.160
Gizzard	1.57±0.10	1.65±0.10	0.648
Abdominal fat	2.02±0.31	1.92±0.30	0.632
	Relative cut yields		
	(% proportion of carcass)		
Breast	20.64±0.94	21.77±0.78	0.379
Thigh	17.19±0.78	17.63±0.45	0.638
Drumstick	12.19±0.37	12.39±0.40	0.726
Wings	11.87±0.11	11.67±0.15	0.302
Trunk	38.12±1.31	36.55±1.02	0.368

TM – group fed with feed mixture for three phases, similar to slaughter turkeys, CM – group fed with feed mixture dedicated for two phases, similar to broiler chickens.

Carcass characteristics of guinea fowl are shown in Table 3. Relative carcass and organ yields were not different between the groups although carcass, edible giblets, breast, and thigh yields were numerically better in CM group.

### 3.2 Meat quality

Table 4 details the breast and thigh muscle quality of guinea fowl fed commercial diets under dedicated feeding programs. Technological traits of breast and thigh meat were not affected by different diets. The results were pointed at proper post-mortem glycolysis. Although any differences were not confirmed statistically, it is visible that numerically slightly better traits linked with water-holding capacity or tenderness were characterized in the breast meat birds in CM group. However, breast and thigh muscles of birds from the CM group diet were characterized by lower $L^{*}$ and $a^{*}$ values compared to the TM group.

**Table 4:** Breast and thigh meat quality of guinea fowl in response to diets based on nutrient specifications for three- or two-phased diets (mean±SEM).

Item	Breast meat quality			Thigh meat quality		
	TM	CM	P value	TM	CM	P value
${\mathrm{pH}}_{15}$	5.61±0.19	5.99±0.21	0.804	5.94±0.13	6.01±0.06	0.062
${\mathrm{pH}}_{60}$	5.76±0.09	5.77±0.13	0.040	5.76±0.04	5.80±0.07	0.088
${\mathrm{pH}}_{24h}$	5.48±0.07	5.45±0.06	0.852	5.64±0.07	5.56±0.06	0.414
$L^{*}$	54.78±1.02	54.47±0.97	0.964	45.46±0.89	45.35±0.64	0.339
$a^{*}$	3.08±0.63	2.18±0.41	0.161	10.01±0.56	9.75±0.83	0.447
$b^{*}$	10.24±0.51	10.26±0.56	0.774	9.95±0.52	9.33±0.42	0.347
Natural loss (%)	1.55±0.22	1.94±0.20	0.578	0.72±0.22	1.12±0.35	0.210
Cooking loss (%)	16.95±1.56	15.79±1.57	0.613	21.08±1.92	20.48±2.84	0.865
Water-holding capacity (${\mathrm{cm}}^{2}$)	64.14±2.57	63.22±2.65	0.807	62.45±1.66	60.83±2.47	0.597
Shear force (N)	14.22±1.17	12.66±0.98	0.467	19.95±1.43	21.00±2.71	0.373

TM – group fed with feed mixture for three phases, similar to slaughter turkeys, CM – group fed with feed mixture dedicated for two phases, similar to broiler chickens.

### 3.3 Fatty acid profile

The fatty acid profile of thigh and breast muscles of guinea fowl fed commercial turkey and broiler diets is presented in Tables 5 and 6. Most of fatty acids were not affected by different diets. Polyunsaturated fatty acids such as γ-linolenic acid (GLA) and alpha-linolenic acid (ALA) were higher (P<0.05) in breast meat of birds in the TM group; however, meat from the CM group contained numerically more arachidonic acid (AA) in breast meat. In addition, the CM group had a numerically higher content of polyunsaturated fatty acid (PUFA) in breast meat. At the same time, values of PI were smaller in both muscles, which points towards the longer shelf life of this meat. Other indexes of fatty acid profile were similar between the groups regardless of muscle type. Any diet used did not contribute to an increase in the hypercholesterolemic properties of meat.

**Table 5:** The content of particular fatty acids in breast and thigh meat of guinea fowl in response to diets based on nutrient specifications for three- or two-phased diets (mean±SEM).

Item	Breast meat			Thigh meat		
	TM	CM	P value	TM	CM	P value
C 12 : 0	0.143±0.011	0.167±0.015	0.326	0.110±0.007	0.130±0.004	0.034
C 14 : 0	1.127±0.050	1.110±0.032	0.783	1.023±0.029	1.087±0.018	0.097
C 15 : 0	0.140±0.007	0.143±0.009	0.782	0.467±0.207	0.137±0.002	0.171
C 16 : 0	26.23±0.440	25.02±0.528	0.108	25.75±0.091	25.88±0.302	0.687
C 17 : 0	0.190±0.010	0.187±0.011	0.826	0.207±0.017	0.213±0.015	0.778
C 18 : 0	7.693±0.603	8.593±0.347	0.225	8.250±1.059	9.353±0.652	0.400
C 20 : 0	0.133±0.008	0.167±0.015	0.089	0.123±0.009	0.147±0.012	0.149
C 22 : 0	0.130±0.020	0.170±0.029	0.275	0.117±0.030	0.157±0.028	0.346
C 24 : 0	0.100±0.000	0.110±0.000	tr	0.060±0.000	0.045±0.009	0.312
C 14 : 1 n5	0.340±0.055	0.287±0.018	0.388	0.317±0.061	0.273±0.026	0.538
C 16 : 1 n7	5.663±0.739	4.747±0.303	0.291	5.963±0.923	5.093±0.464	0.419
C 17 : 1 n7	0.130±0.010	0.093±0.012	0.037	0.147±0.012	0.143±0.008	0.816
C 18 : 1	30.44±1.014	30.50±1.191	0.973	30.08±0.801	30.11±0.909	0.981
C 20 : 1	0.540±0.029	0.577±0.046	0.515	0.473±0.040	0.507±0.019	0.475
C 22 : 1 n9	tr	tr	tr	0.070±0.000	tr	tr
C 18 : 2 n6 (LA)	21.87±0.433	22.46±0.617	0.452	22.97±0.207	22.37±0.448	0.263
C 18 : 3 n6 (GLA)	0.123±0.008	0.100±0.004	0.040	0.143±0.020	0.110±0.010	0.178
C 18 : 3 n3 (ALA)	1.060±0.015	0.953±0.040	0.032	1.080±0.080	0.963±0.053	0.250
C 20 : 2 n6	0.680±0.158	0.887±0.265	0.130	0.427±0.035	0.427±0.035	1.000
C 20 : 3 n6	0.230±0.044	0.253±0.056	0.750	0.237±0.008	0.237±0.018	1.000
C 20 : 4 n6 (AA)	2.680±0.802	3.117±0.846	0.716	2.017±0.346	2.180±0.398	0.763
C 20 : 5 n3	0.130±0.000	0.160±0.000	tr	0.055±0.003	0.055±0.003	1.000
C 22 : 2 n6	0.180±0.000	0.360±0.000	tr	0.180±0.035	0.123±0.021	0.172
C 22 : 6 n3 (DHA)	0.253±0.076	0.270±0.093	0.561	0.220±0.020	0.157±0.022	0.061

TM – group fed with feed mixture for three phases, similar to slaughter turkeys, CM – group fed with feed mixture dedicated for two phases, similar to broiler chickens, tr – trace (content lower than 0.05 %); C14 : 0 – myristic acid, C15 : 0 – pentadecylic acid, C16 : 0 – palmitic acid, C17 : 0 – margaric acid, C18 : 0 – stearic acid, C20 : 0 – arachidic acid, C22 : 0 – behenic acid, C24 : 0 – lignoceric acid, C14 : 1 – tetradecenoic acid, C16 : 1 – palmitoleic acid, C17 : 1 – heptadecenoic acid, C18 : 1 – oleic acid, C20 : 1 – gondoic acid, C22 : 1 – nervonic acid, C18 : 2 n6 – linoleic acid (LA), C18 : 3 n6 – γ-linolenic acid (GLA), C18 : 3 n3 – α-linolenic acid (ALA), C20 : 2 n6 – eicosadienoic acid, C20 : 3 n6 – dihomo-γ-linolenic, C20 : 4 n6 – arachidonic acid (AA), C20 : 5 n3 – eicosapentaenoic acid, C22 : 2 n6 – docosadienoic acid, C22 : 6 n3 – docosahexaenoic acid (DHA).

**Table 6:** Fatty acid profile in breast and thigh meat of guinea fowl in response to diets based on nutrient specifications for turkeys and broilers (Mean±SEM).

Item	Breast meat			Thigh meat		
	TM	CM	P value	TM	CM	P value
SFA	35.80±0.17	35.53±0.19	0.304	35.72±1.01	37.13±0.49	0.248
MUFA	37.10±1.59	36.19±1.26	0.662	37.02±1.52	36.13±1.24	0.661
PUFA	26.98±1.48	28.19±1.40	0.564	27.14±0.53	26.60±0.86	0.599
n3	1.35±0.09	1.27±0.09	0.524	1.34±0.07	1.15±0.03	0.059
n6	25.63±1.39	26.93±1.34	0.516	25.81±0.60	25.44±0.86	0.735
n9	30.75±1.00	30.83±1.19	0.962	30.42±0.77	30.45±0.89	0.978
n6/n3 ratio	19.01±0.22	21.46±1.16	0.066	19.79±1.71	22.13±0.95	0.258
PUFA/SFA ratio	0.75±0.04	0.79±0.04	0.484	0.76±0.01	0.72±0.02	0.057
PI	39.37±4.54	53.15±4.03	0.161	37.41±1.42	36.89±2.04	0.837
AI	0.48±0.01	0.46±0.01	0.161	0.47±0.00	0.48±0.00	0.020
TI	0.99±0.01	0.98±0.01	0.659	0.99±0.05	1.06±0.02	0.225
DFA	71.78±0.47	72.98±0.52	0.118	72.41±0.14	72.08±0.68	0.292
HFSA	27.50±0.50	26.29±0.55	0.133	26.88±0.31	27.10±0.77	0.535
h/H	2.02±0.00	2.10±0.00	0.955	2.00±0.01	1.97±0.04	0.424

TM – group fed with feed mixture for three phases, similar to slaughter turkeys, CM – group fed with feed mixture dedicated for two phases, similar to broiler chickens, SFA – saturated fatty acid, PUFA – polyunsaturated fatty acid, MUFA – monounsaturated fatty acid, PI – peroxidation index, AI – atherogenic index, TI – thrombogenic index, DFA – desirable fatty acid, HFSA – hypercholesterolemic saturated fatty acid, h/H – hypocholesterolemic / hypercholesterolemic ratio.

## 4 Discussion

The given body weight significantly exceeds the value obtained in the experiment of Khairunnesa et al. (2016) in the 14th week of life of guinea fowls of the pearl variety. The authors described the birds' rearing system as an intensive one and used feed with 21 % protein content. Wallace et al. (2018) reported that the body weight of 18-week-old guinea fowl ranged from 1058 to 1224 g regardless of the protein source used in the feed (insect larvae vs. fish meal), which was about 50 % of the final body weight obtained in this study. Such a huge difference may be mainly due to the fact that the laying-type variety of guinea fowl was used in addition to nutrient content of diets such as the protein level that remained around 15 %. The results obtained in terms of feed intake are more than 1 kg better (average value for 14 weeks) than those noticed by Bernacki et al. (2012a) regardless of the evaluated color variety of birds. However, it should be considered that the distinguishing trait was the utility type of birds (meat vs. laying) apart from the plumage color. The meat-type guinea fowls were used in the study of Tufarelli et al. (2015) by replacing soybean in the feed mixture with micronized dehydrated lupine. At the age of 12 weeks, they reached a weight of over 1900 g, which corresponds to the guinea fowls of the TM group in this study. Birds fed commercial broiler diets were slightly lighter. The growth curve for meat-type French guinea fowl depending on sex is presented by Nahashon et al. (2006). They reported that the average body weight of 9-week-old male and female birds should be 1312 and 1304 g, respectively. The results obtained for guinea fowls included in our experiment were significantly better despite lower protein content in feed. Baeza et al. (2001) indicated the importance of bird genotype (growth rate), sex, and thermal conditions during rearing for this trait. Females were bigger irrespective of the genotype as well as high productive line of birds kept during the winter period and with a standard growth rate. In this study, the influence of sex on body weight was not considered, and temperatures that were standardized can be omitted; therefore, the results are in line with those of Baeza et al. (2001). However, FCR was considerably bigger in our study, by about 800 in the TM group and over 1000 g for guinea fowl in the CM group, which might have resulted from the lower protein content of commercial diets used.

Agwunobi and Ekpenyong (1991) considered the rearing efficiency of broiler chickens and guinea fowls after 12 weeks of rearing using the commercial diets for chickens. The most important observation was a significantly higher survival rate of guinea fowl in comparison with chickens. They also showed by almost 50 % lower feed intake along with higher FCR (4.6 vs. 3.4 $\mathrm{kg}\;{\mathrm{kg}}^{-1}$ of body weight gain) resulting from lower final body weight (1.4 vs. 3.4 kg). It seems that the results of this study can be considered similar to those quoted; however, the livability of birds included in this study amounted to 100 % regardless of the group. Premavalli et al. (2013) justified the use of broiler diets in guinea fowl production due to extensively rearing of these birds and lower protein requirement in extensive rearing systems. Consequently, feed with higher nutritional value may contribute to better rearing results. Yamak et al. (2018) demonstrated a significant impact of the housing system on the body weight of meat-type guinea fowl. Greater body weight was observed in birds kept without access to runs than in a free-range system. However, this was much lower than that obtained in our study. FCR was similar to that obtained in the TM group. The degree of feed conversion largely determines the cost of rearing the birds. In guinea fowl, this ratio is quite high, exceeding 4 $\mathrm{kg}\;{\mathrm{kg}}^{-1}$ at 14 weeks of rearing (Bernacki et al., 2012a). Similar values in intensive rearing can generate significant feeding costs, thus limiting the meat-type guinea fowl rearing on a wider scale.

The TM or CM diets used in this experiment did not affect the body weight of birds including the slaughter weight. The obtained carcass yield was similar to that shown by Ebegbulem and Asuquo (2018) for 14-week-old black and pearl guinea fowls and their crosses. Bernacki et al. (2012b) found that the color variety of birds can modify the body weight and parts' yield; considerably better values of these traits were observed in birds of the gray variety. However, the slaughter weight of guinea fowl recorded in our study at the end of the 14th week was about a kilogram higher than in the cited studies, with a much better slaughter performance (the difference amounted to 8 %) but with a lower yield of pectoral muscle in carcasses (6 %). Similar comparison was seen between the results of our study and those of Kokoszynski et al. (2011). Body weight and slaughter performance were much lower, whereas breast yield exceeded 23 %. An extremely high yield of pectoral muscles (about 30 %) in the carcasses of guinea fowl kept in Sudan was recorded by Eltayeb et al. (2015), but they indicated that the diversity of both production and slaughter performance parameters including the weight of giblets is even due to the flock location within a single country, which is probably associated with different thermal conditions. Teye et al. (2001), while comparing the slaughter parameters of guinea fowl of different origins (imported and local to Ghana), found that imported birds had a higher body weight and carcass yield than the local variety in addition to higher edible giblet yields. It is noteworthy that relative gizzard weight to that of carcass was ≈8 % in our studies; after conversion, the relative gizzard yield was about 2 %. It probably resulted from the contribution of green fodder in the feeding (Batkowska et al., 2015).

It is well known that the animal diet is a key factor in determining the quality of meat. Quantifiable properties of meat such as water-holding capacity, shear force, drip loss, cooking loss, pH, and color are indispensable for processors to acquire functional properties that will ensure a final product characterized by a quality acceptable to the consumers (Cheng and Sun, 2008). Water-holding capacity of fresh meat is an important quality attribute since it affects major characteristics of the cooked meat such as technological and eating quality. Drip loss as a result of postmortem myofibril shortening is caused by pH fall, denaturation of myosin, and the formation of actomyosin at the onset of rigor mortis. Any loss of water or shrinkage due to loss of water hints at a less optimal quality. Excessive shrinkage can have an adverse effect on appearance, juiciness, and tenderness (Bertram et al., 2000). Among many other factors, feeding of animals affects the ability of the animal muscles to retain water (Cheng and Sun, 2008). However, the current study did not show a significant effect of feeding on the water-holding capacity of breast and thigh muscles of meat-type guinea fowls. Among the feeding strategies that have the potential to improve the ability of the animal muscles to retain water, the supplementation of vitamin E or D${}_{3}$ has its influence on drip loss. In present study, although the composition of the mixtures differed in the content of these minerals, this did not affect the water-holding capacity, drip loss, or cooking loss of muscles. Another important factor that affects meat quality and determines consumer preferences is the meat color. Lightness ($L^{*}$) values in the present study ranged between 45.35 and 45.46 for thigh muscle and between 54.47 and 54.78 for breast muscle and were lower than the values that were reported in a previous study (Sarica et al., 2019). In contrast, other researchers obtained lower $L^{*}$ values for guinea fowl muscles (Kokoszyński et al., 2011). Redness ($a^{*}$) values in the present study ranged between 2.18 and 3.08 for breast muscle and between 9.75 and 10.01 for thigh muscle. The values obtained are lower for the thigh muscles and higher for the breast muscles compared to those obtained by Sarica et al. (2019).

Few studies have been published on the quality of guinea fowl meat. To the best of our knowledge, we are the first to investigate the effect of feeding commercial diets to guinea fowl during dedicated different phases of growth on the physicochemical properties of meat. Laudadio et al. (2012) substituted soybeans with peas in the diet of guinea fowl raised for 12 weeks. Despite a slightly lower percentage of protein in the diet compared to current study, the birds achieved a similar body weight, while it was found that the breast muscles were characterized by lower $L^{*}$ parameters, indicating the lightness of muscles. The values of pH and drip loss of muscle were similar to those obtained in this experiment; however, the muscles showed a lower water-holding capacity value compared to our results. Higher water-holding capacity values of more than 10 % were observed for the thigh muscles. Water-holding capacity of the breast muscle was similar to that presented by Kokoszyński et al. (2011), whereas it was higher in the case of thigh muscles than in the cited studies. The research showed that the share of the abdominal fat was 4 times lower than that in the authors' own research, despite a slightly longer rearing (16 weeks). This could indicate a greater amount of intramuscular fat and therefore a greater water-holding capacity. It is particularly interesting because the fatty content of the carcass is linked to the intestinal fat content. Significantly higher water-holding capacity values were recorded in the studies by Sarica et al. (2019), regardless of the breeding system or age of guinea fowl; their muscles, regardless of the type, also lost more water due to natural loss and loss after thermal treatment compared to the muscles of the birds in this study. Musundire et al. (2017) analyzed the influence of age and gender on body weight, carcass characteristics, and physical and chemical properties of breast muscles of chickens and free-range guinea fowl. They found that guinea fowls had a higher body and carcass weight, as well as the breast muscle weight compared to chickens. The cooking loss was greater in guinea fowl, males, and younger (growing) birds than in chickens, female, and adult birds.

The fatty acid profile of breast muscles obtained in our study considerably differs from that presented by Tufarelli et al. (2015). They studied the effects of dietary substitution of soybean meal with micronized-dehulled white lupin in meat-type guinea fowl on meat fatty acid composition. Authors showed lower concentration of saturated (SFA) and higher of unsaturated fatty acids (MUFA and PUFA) in breast muscles compared to our findings. Studies carried out by Chiroque et al. (2018) showed considerably lower n 6 / n 3 values for guinea fowl muscles fed diet having 18.5 % protein compared to our results. Similarly, the content of fatty acids demonstrated in the study performed by Laudadio et al. (2012) was different from that in our study. The content of ALA and DHA in muscles from guinea fowl fed micronized-dehulled pea was higher, while GLA content was almost 2 times lower. Differences may arise from many factors including various ingredients used in the feed or the age of birds. According to Hoffman and Tlhong (2012), the differences may result from the analysis conditions. The quoted study showed a lower content of SFA, higher unsaturated fatty acid (UFA), and greater SFA / UFA ratio than in the current study. The reasons for this may be differences in rearing conditions and the availability of green fodder for birds (they came from small-scale farms). As indicated by Dal Bosco et al. (2016), the share of green fodder in poultry nutrition may modify the fatty acid profile in meat.

A similar proportion of SFA content in the breast muscle of guinea fowls was found by Bernacki et al. (2012b). Their study also showed a higher content of PUFA and a lower content of MUFA. There was a greater percentage of ALA and a lower concentration of DHA. The birds were fed balanced diets for broiler chickens and kept under controlled environmental conditions. It is important to find the differences in the chemical composition of meat, including to some extent the profile of fatty acids due to the color variety of guinea fowls.

An analysis of the available literature indicates significant variability of traits within the species *Numida meleagris*. It results from color variety, growth rate, sex, age, husbandry system, diet, and climatic conditions. Therefore, the problem of the lack of possibility to generalize the obtained research results often arises. Nevertheless, despite their much lower popularity than the typical meat-type poultry species, such as chickens or turkeys, guinea fowls have a great potential not only in terms of the quantity and quality of meat obtained but also as research material, and their advantages in this respect are worth propagating.

## 5 Conclusion

The presented results confirm the thesis related to the benefits of feeding meat-type guinea fowl with commercially available diets for other meat-type poultry species. Irrespective of the diet, comparable values were obtained among others in terms of the body weight and slaughter performance. Various protein content in both diets did not influence the above-mentioned features. A detailed analysis of technological traits of the pectoral and thigh muscles indicates that a similar quality of the final product was obtained, which may indicate the universality of commercially available diets in the market for rearing the meat-type guinea fowl without any loss of production. The difference in favor of TM diet, with a higher protein content, concerned the feed conversion ratio, but for economic reasons (lower protein diets are cheaper) it seems more advantageous to feed guinea fowl with CM diets.

## Data Availability

Data used and analyzed during this study are available from the corresponding author upon reasonable request.
